# Understanding Older Adults’ Experiences With Technologies for Health Self-management: Interview Study

**DOI:** 10.2196/43197

**Published:** 2023-03-21

**Authors:** Elsy Paola Garcia Reyes, Ryan Kelly, George Buchanan, Jenny Waycott

**Affiliations:** 1 School of Computing and Information Systems Faculty of Engineering and Information Technology The University of Melbourne Melbourne Australia

**Keywords:** older adults, technology, health self-management, motivator, enabler, barrier

## Abstract

**Background:**

Many older adults now use technologies such as wearable devices and telehealth services to support their health and well-being while living independently at home. However, older adults vary in how they use these technologies, and there is a lack of knowledge regarding the motivations that influence their acceptance and use of health-related technologies in home environments.

**Objective:**

This study aimed to understand the types of technologies that older adults use to support their health and the factors that motivate them to use their chosen technologies to support their health. In addition, we aimed to understand the factors that enable the effective use of technologies for health self-management and to identify the barriers that can negatively affect the adoption of technologies.

**Methods:**

A total of 22 older adults participated in semistructured interviews regarding their experiences of using technologies for health self-management. Interview transcripts were analyzed through an in-depth thematic analysis.

**Results:**

The interviews revealed that a range of technologies, such as videoconferencing software, fitness trackers, and other devices, were being used by older adults to support their health. Interviews showed that participants were motivated to use technologies to monitor health issues, to stay active and connected, and to record and change their behavior in the light of foreseen risks related to their future health status. Enablers that facilitated the effective use of technologies include social and organizational influence, convenient access to health care and safety provided by the technology, and easy setup and low cost of the technology. Barriers include information overload and a sense of futility about future health decline; telehealth being an inadequate substitute for in-person consultation; concerns about trust related to privacy and accuracy; and technologies being stigmatizing, uncomfortable to use, expensive, and unfamiliar.

**Conclusions:**

This study suggested that older adults were using a variety of technologies to prevent or prepare for future health decline, evidencing a resilient attitude toward health and aging. In addition, older adults were willing to continue using the technology when there was a perceived need. The enabler mentioned by most participants was the social and organizational influence that included health care staff, family, friends, and organizations. This analysis provides a better understanding of how older adults use technologies to support their health and can guide the provision of appropriate health technologies for them.

## Introduction

### Background

Information and communication technologies have considerable potential to support older adults in accessing health care services and in self-managing their health. Recently, the COVID-19 pandemic has accelerated the interest in supporting older adults’ health and has emphasized the critical importance of self-monitoring as a facet of public health [1]. Self-management refers to an individual’s ability to manage symptoms; treatments; lifestyle adjustments; and the psychosocial, cultural, and spiritual consequences of health conditions, specifically chronic diseases, in collaboration with family, community, and health care professionals [2]. Examples of technologies for health self-management include wearable devices, telecare, sensor-based monitoring systems, and mobile apps. These technologies can help older adults to control their diet and physical activity or achieve emotional self-control [3-5]. Such technologies, if used effectively, could increase the efficiency of health care, reduce the workload for providers, reduce medical costs, and improve older adults’ well-being [6]. Furthermore, these technologies can enable older adults to maintain their autonomy and independence as they age [7-9].

Recent research related to technology adoption among older adults has indicated that older adults are increasingly familiar with digital technologies [10-12] and are interested in using technology to manage their health, such as measuring heart rate, keeping an activity diary, and monitoring stressful situations [13,14]. However, older adults’ experiences with technology can vary greatly, and many factors can influence whether an older adult successfully uses technologies for health self-management. This means that there is reason to be cautious about predicting the rising uptake of health technology by older adults. Previous quantitative studies have shown that older adults have reservations about using health-related technologies to obtain health information and advice [15,16]. However, these studies were conducted between 3 and 6 years ago, and the situation could have changed in recent years, as these technologies have become more commonplace.

Other studies suggest that there are a range of factors that affect the adoption of health technologies by older adults. Researchers have investigated older adults’ use of activity trackers and sleep monitoring devices [13,14,17-20]. Although there is a positive interest in using such technologies, encouraging their ongoing use is a challenge [19], and social support has been identified as the main motivation for encouraging the use of these technologies in long-term users [14]. In addition, studies have concluded that the design and use of wearable devices and mobile apps must consider age-related cognitive, sensory, and motor function changes in the older generation to ensure the adoption of these technologies by them [13,20].

We also understand some of the potential applications of this technology. A key principle is supporting aging in place, where older adults are supported in living independently in their established home and community, rather than moving to specialist accommodation [21]. Previous studies have identified possible barriers to technology adoption for aging in place. These barriers include device usability, accessibility, reliability, affordability, and privacy [22,23]. Concerns about trust related to privacy and security, stigma, the lack of control over technology, the lack of human response, inaccuracy, need for training, and anxiety are further impediments [24,25]. A comprehensive review of smart residential environments added that security, the lack of interoperability, complexity, and the lack of perceived utility were concerns of older adults [7]. Almathami et al [26] conducted a systematic review of the factors that influence the use of web-based home health consultation systems or telemedicine health services. They identified internal factors, including users’ behaviors and motivations while using and interacting with the system and patients’ beliefs and perceptions of the relative advantages and disadvantages of the web-based home health consultation system.

The role of assistive technology in supporting the health of older adults has also received attention. Greenhalgh et al [27] conducted a study with 40 participants to develop a theoretical model of assistive technology use. They found that telehealth and telecare seldom met older adults’ needs and did not assist them to live with an illness. Yusif et al [28] conducted a systematic review of factors that concern older adults in their decision to adopt assistive technology. They identified privacy as the main concern to older adults, followed by trust, the lack of functionality or added value, financial cost, and the ease of use. Other factors that negatively affect technology adoption included the suitability for daily use, perception of no need, stigma, fear of dependence, and lack of training.

These studies have collectively focused on why older adults have hesitated to or have been prevented from using technologies before or after they are familiar with them. However, the studies did not examine the motivations of older adults for using these technologies. This study closes this gap. It takes a broad perspective, looking at a variety of technologies that participants identify as relevant to their health self-management. This perspective provides a vital understanding of older adults’ needs and, consequently, can improve the use of technologies and care for older adults.

### Objectives

This study aimed to understand the technologies that older adults use to support their health and the factors that motivate them to use technologies to support their health. In addition, this study aimed to understand the factors that enable the effective use of technologies for health self-management and to identify the barriers that can negatively affect technology adoption. We adopted a qualitative approach involving semistructured interviews with 22 participants.

Understanding the motivators, enablers, and barriers that affect technology-based health management is essential to ensuring that future technologies are designed and deployed appropriately. To be effective, technologies must be designed to align with health self-management at a time of life when good health is not guaranteed.

## Methods

### Ethics Approval

All procedures were approved by the Human Research Ethics Committee of The University of Melbourne (ID# 1955800).

### Participants and Recruitment

We recruited participants by contacting organizations that provide services to older adults who live independently at home. The recruitment criteria included individuals who were aged >65 years; lived in Melbourne (Australia); used a device for their health care (eg, home blood pressure monitor, blood glucose monitor, fitness tracker, etc); and spoke English.

A total of 5 organizations distributed information about our research to their members. Respondents who registered their interest in the study were then contacted, and an interview was arranged. A total of 22 participants agreed to be interviewed. Drawing on the concept of “information power,” this sample size was deemed sufficient [29]. Using information power as a guiding principle to assess the appropriate sample size in qualitative research gives weight to criteria such as clearly defined aims, sample specificity (eg, older adults who use technologies for health self-management), and the quality of conversations with interviewees. Our study met these criteria; therefore, we are confident that a sample size of 22 is sufficient to gain in-depth insights into the phenomenon studied. Furthermore, this sample size is in line with guidelines based on a meta-analysis of typical sample sizes required to reach theoretical “saturation” in qualitative data [30], and the study found that saturation is typically reached with between 8 and 17 interviews.

All the interviews were conducted from October 2020 to January 2021. To protect their anonymity, all the participants have been given a pseudonym and are referred to by their pseudonym in the *Results* section.

### Procedure

Participation was voluntary, and the participants did not receive compensation in the study. Participants read a plain language statement and signed a consent form. All interviews were conducted by author EGR. The interviews were held via phone or videoconference, using WhatsApp (Meta Platforms Inc), FaceTime (Apple Inc), or Zoom (Zoom Video Communications), from October 2020 to January 2021. We conducted interviews to gain an in-depth understanding of older adults’ motivations and experiences that affect their acceptance and use of technologies for health self-management. The interviewer asked questions related to the acquisition and use of technologies that participants used to manage their health, questions about participants’ motivations for using these technologies, who influenced participants’ decisions to use these technologies, and what they did not like about these technologies.

### Data Analysis

Each interview was audio recorded following the participant’s approval. Then, the interviews were transcribed verbatim and analyzed using thematic analysis [31]. The thematic analysis involved the following six stages: (1) becoming familiar with the data, (2) generating codes, (3) generating initial themes, (4) reviewing themes, (5) defining and naming themes, and (6) producing the report. The data were coded by EGR using an inductive approach. Preliminary codes, subthemes, and key themes were refined through discussions among the research team. NVivo (version 12; QSR International) software was used to identify and categorize the codes and organize the data.

## Results

### Participants

A total of 22 people agreed to be interviewed, including 14 (64%) women and 8 (36%) men. Participants’ age ranged from 65 to 87 (mean 73, SD 6) years. Most participants (n=20, 91%) were educated beyond secondary school. A total of 14 (64%) participants were living with their partner, and 8 (36%) participants were living alone. Additional demographics are summarized in Table 1. Multimedia Appendix 1 provides more details about the interviewees.

**Table 1:** Participant demographics (N=22).

Demographics			Value
Age (years), mean (SD)			73 (6)
**Sex, n (%)**			
	Female	14 (64)	
	Male	8 (36)	
**Highest level of education, n (%)**			
	Tertiary institution, university, or other higher educational institution	20 (91)	
	Secondary school	2 (9)	
**Marital status, n (%)**			
	Married	13 (59)	
	Widowed	4 (18)	
	Single	3 (14)	
	Divorced	1 (5)	
	Prefer not to answer	1 (5)	
**Employment, n (%)**			
	Retired	19 (86)	
	Semiretired	2 (9)	
	Working part time	1 (5)	
**Household composition, n (%)**			
	Live with his or her partner	14 (64)	
	Live alone	8 (36)	
**Need for help or supervision, n (%)**			
	No	17 (77)	
	Yes, sometimes	5 (23)	
**Reasons for help or supervision, n (%)**			
	No need for help or supervision	17 (77)	
	Long-term health condition	3 (14)	
	Short-term health condition	1 (5)	
	Other cause or community services come in to clean	1 (5)	

### Types of Technologies Used

Participants mentioned a range of technologies they used to support their health activities at home. These included videoconferencing software and phone devices to access health care services (telehealth), wearable devices to monitor activity and health status, personal alarm systems to alert carers in the case of emergencies, web-based sources for health information, and a range of other specific health technologies.

Telehealth, a remote health care service, was the technology used by most respondents (14/22, 64%). To access telehealth, the participants used videoconferencing software or phone calls.

Among the wearable devices mentioned by the 22 respondents were fitness trackers (n=8, 36%) and personal alarm systems such as pendant alarms (n=6, 27%). In addition, the participants mentioned using medical devices such as blood pressure monitors (n=9, 41%), blood sugar or glucose monitors (n=4, 18%), hearing aids (n=5, 23%), a pulse oximeter that measures blood oxygen level and pulse rate (n=1, 5%), a continuous positive airway pressure (CPAP) machine that treats sleep apnea disorders (n=1, 5%), an implantable cardiac defibrillator and pacemaker (n=1, 5%), and a pelvic floor stimulator that strengthens the pelvic floor muscles to reduce incontinence (n=1, 5%).

Some participants identified mobile devices (n=4, 18%) and personal computers or laptops (n=3, 14%) as technologies they used to look after their health. Moreover, 6 (27%) participants were using apps related to food and physical activities.

All the participants (22/22, 100%) had access to the internet, and 5 (23%) of them commented that they used the internet to search websites for information related to their health.

Other technologies mentioned by the participants included Google Calendar, used by 1 (5%) respondent as a diary to remember medical appointments and activities, and a customized spreadsheet, used by another participant (n=1, 5%) for health monitoring and tracking. A summary of these technologies is presented in Table 2.

**Table 2:** Types of technologies used to support participants’ health activities at home (N=22).

Types of technologies		Participants, n (%)
**Remote health care service**		
	Telehealth	14 (64)
**Wearable devices**		
	Fitness tracker	8 (36)
	Personal alarm system	6 (27)
**Medical devices**		
	Blood pressure monitor	9 (41)
	Hearing aids	5 (23)
	Blood sugar or glucose monitor	4 (18)
	Pulse oximeter	1 (5)
	CPAP^a^ machine	1 (5)
	Implantable cardiac defibrillator and pacemaker	1 (5)
	Pelvic floor stimulator	1 (5)
**Others**		
	Apps related to food and physical activities	6 (27)
	Mobile device	4 (18)
	Personal computer or laptop	3 (14)
	Google Calendar	1 (5)
	Customized spreadsheet	1 (5)

^a^CPAP: continuous positive airway pressure.

### Motivators: Factors That Motivate the Use of Technologies for Health Self-management

#### Overview

We present 3 themes that provide insight into why participants were using technology to manage their health. First, many participants were motivated by the need to keep track of health-related information to manage chronic health conditions, such as diabetes, and to stay independent. Second, participants were motivated to use technology to stay active and socially connected and saw this as an important part of their health self-management. Third, some participants were motivated to use technologies to monitor their health and change their behavior because of prior knowledge and awareness of personal risks related to their future health decline.

#### Theme 1: Monitoring Chronic Health Issues to Stay Independent

Of the 22 participants, 14 (64%) said that their main motivation for using technology was to monitor their health issues. Within this group, 12 (86%) participants reported that the technology was useful for monitoring or helping manage existing medical conditions or disabilities. For example, 1 (7%) participant used an app to track vital signs and blood pressure that could affect his health and to be in contact with his physician (general practitioner):

I have an app on my phone that is connected to my GP and so my cholesterol levels, my heart rate, my weight, blood pressure, are all communicated back to him. If I enter a value in here, it shows up on his screen and if he’s concerned he will text me to come and see him or just...It will text me to make an appointment. In fact, I got one earlier this week where he said, it’s time for your skin check. So, I get the skin check. I’ll take that phone off.

P2, Bob

Furthermore, 3 (21%) participants commented that if they did not monitor their health issues, they would lose their capabilities and risk losing their independence, as described by Nancy:

I think I’m going to end up with knee surgery, probably a knee replacement. I’ve had minor knee surgery because I also tore a cartilage doing some stuff, but I think I’m going to end up with a knee replacement. Health, I’m going to have to keep control of diabetes because I know otherwise my independence, if I would lose my vision or if my kidneys start failing, that’s going to really affect again my independence. So, I’ve got a handle on the diabetes so that the...That just gets harder as the system gets older so I really, really got to work on that.

P14, Nancy

In addition, 3 (21%) participants wanted to be healthy to avoid being a burden to their family. A participant commented the following:

Some people would say money is more important. Some people would say family is more important, but I figure, no matter how wealthy you are, if your health is rotten, money means nothing. And if put family first and your healthy is no good, you are a burden on your family and they look after you. So you have to be healthy so you can do what your family might need regardless of your age.

P11, Katy

Participants reported that technologies such as telehealth and wearable and medical devices were useful for monitoring or assisting with medical conditions or disabilities. These findings suggest that these health technologies fulfill the purpose for which they are designed and help older adults maintain their independence by monitoring and managing their health.

#### Theme 2: Staying Active and Connected

Most of the participants performed technology-supported activities that helped them stay physically or mentally active and connected with family and friends. Overall, 14 (64%) of the 22 participants used technologies that were aligned to their objectives to stay active. For example, one participant commented as follows:

I have a Fitbit, which I carry in a pocket all the time. I aim for 10,000 steps, but don’t often get there since I had the problem with the disk [back injury]. But I’m usually over 2000 or thereabouts. Sometimes higher. I do tend to sit. We also have weighing scales that are connected to the Fitbit. So I keep track of my weight, and also the percent body fat. And I think that’s it.

P13, Michael

Of the 22 participants, 5 (23%) who were using apps related to fitness and food, videoconferencing software, and hearing aids mentioned that they shared information related to the progress and goals of their activities with relatives and friends. This helped them to stay socially connected:

The Strava app is a really good app because it links you with a group of friends, people you know who are also doing something similar so if you can’t meet in person, you can sort of meet with your chat on the Strava app after you’ve recorded a ride. And, people give you positive comments, or they talk about the photos, or they say, well done for the distance or gee, that was a lot of mountain climbing you did today. So, you get a lot of positive feedback from your friends so I think that’s a very positive thing.

P7, Gwen

One participant who had a hearing impairment and was using hearing aids to go out, talk to people, or watch television mentioned that hearing aids helped her avoid isolation:

Yes. I’d say that they’re very important. The hearing aids are especially important, otherwise you’re isolated from people and can’t understand what they’re saying…

P3, Cyndi

Of the 22 participants, 3 (14%) reported that these technologies helped them perform regular activities. One participant used a Google Calendar to plan his daily activities:

And I have diary that is on Google. I find that very useful to plan my appointments and things like that. Doctors’ appointments, that sort of thing. And to make sure that I don’t forget things that I need to do. I think that’s pretty important.

P6, Frank

#### Theme 3: Knowledge of Risk of Future Health Decline

Of the 22 participants, 10 (45%) demonstrated an awareness of risk to certain diseases, especially because of known genetic traits in the family. On the basis of this knowledge, participants tried to prevent or monitor diseases they suspected were more likely to develop. One participant spoke about how his family history of stroke meant he had increased motivation to monitor his blood pressure, leading him to adopt a blood pressure monitor:

Well, currently, I’m undergoing no major medical treatment so that will be the first thing. I do take blood pressure medication and that type of thing, so my blood pressure is actually controlled, fortunately. Obviously that may have contributed to my father’s, maybe even my grandfather’s stroke. So it’s been very important for me to keep my blood pressure under control. So that means taking the proper medication and getting it reviewed regularly.

P6, Frank

Within this group, 2 (20%) out of 10 participants reported familiarity with health self-management technologies because their close relatives used them to monitor their own medical conditions. For example, one participant commented as follows:

Well, I guess, my late wife had high blood pressure, and she had the monitor to keep a check on that, and so I’d use it [blood pressure monitor] occasionally, just more or less for a bit of fun and see what the readings were.

P10, John

In addition, 3 (30%) participants believed that they had a duty to know about their own health. A participant was motivated to use blood pressure and blood glucose monitors so that she could control her health:

Sometimes people get frightened because they think of bad news. I’ve heard of people who won’t get tested for diabetes in case they have to stop eating cake. I think, would you rather be blind? So, yes, personally I like to know. The things I can control I would rather control, so I’d rather have the knowledge. That’s important except, as I said, there’s only so many things that you can twitch about.

P14, Nancy

### Enablers: Factors that Enable the Use of Technologies for Health Self-management

Our analysis revealed 3 factors that enable the positive uptake of technologies for health self-management. These include social and organizational influence, convenient access to health care and safety provided by the technology, and easy setup and low cost of the technology.

#### Theme 4: Social and Organizational Influence on Decisions to Use Technology

Social and organizational influence was identified as an enabler in the use of technologies for health self-management. All 22 (100%) participants mentioned that health care staff, family, friends, or organizations influenced their use of technologies.

##### Health Care Staff

Of the 22 participants, 11 (50%) mentioned that health care staff recommended or suggested using technologies for health self-management to prevent, monitor, and treat health issues. The participants could monitor their health and report the readings from the device to the medical staff if the results were not within the normal range. A participant described several different technologies recommended by the medical staff:

Well, that [related to the CPAP machine] was a specialist recommendation. But I guess I use it differently to how some people might use it. I extract the card and I read the data on it and check the progress. I don’t bother going back to the sleep physician. I’ve actually got training treatment of sleep disorders, so I know what the data means and if something’s not looking right, then I’ve got a reasonably good idea about what I need to do about it. So that was that was specialist advice. Hearing aids, I’ve had a hearing impairment since childhood. But I only was aided once I was about 40. So again, that was kind of consultation with a health professional that says this could be helpful for you. Blood pressure monitor again,...I suppose, it’s medical advice which says this would be a good idea, or I recommend this or something like that. Yeah.

P15, Olivia

##### Family and Friends

Overall, 3 (14%) participants reported that supportive family members helped them use the technologies. The family members made it easier for older adults to use technologies. A participant said that she received help from her family to use the blood pressure monitor:

My husband has to get it [blood pressure machine] out of the cupboard for me. He usually helps me put it on my arm. I can do that myself, but it’s just easier if he does it, and he knows how to line it up or that sort of stuff. One of my daughters is a registered nurse and she lives not far from me. There have been times when I haven’t been feeling good and she’s come to my house. She can do those readings easily. It’s generally family members who use the machines, but I will do it myself as long as I can get access to it, even if it’s tucked away in a drawer or something, I can usually get it out when I need to.

P17, Rita

Furthermore, 3 (14%) participants received technologies as gifts from their families, and 5 (23%) participants acquired the technologies because their family or friends were using them. A participant started to use an app because her husband was using it:

Because he was already on Strava and I could see how much enjoyment he was getting out of it, and so after a little while I decided to join as well and I enjoy it so much that I probably do more recording than he does.

P7, Gwen

Another participant was influenced by her friends to obtain a fitness tracker because they used fitness trackers to monitor their activities:

I guess the Fitbit, friends had them. And I thought, ‘Oh, that sounds like a good idea, to monitor your activity.’ And it does give you encouragement. And I don’t participate in groups, this is just me looking at what I do.

P12, Linda

##### Organizations

In addition, 5 (23%) participants were using technologies such as apps related to food, blood glucose monitors, hearing aids, or a pendant alarm based on information received from organizations. These were nonprofit organizations that cater to people aged >50 years and public and private organizations that provide assistance with conditions such as diabetes, tinnitus, hearing loss, or dementia. In some cases, these organizations allowed their clients to trial their technologies before purchasing them:

Well, with Academic Hearing, I went there, and they asked me if I’d trial their hearing aids, and I trialled them, and they said yes I could have them.

P19, Tina

Moreover, 7 (32%) participants had the cost of the devices subsidized by the government, care organizations, or health insurance companies. A participant said the following:

There’s an organisation in Australia, Diabetes Australia and a lot of things are subsidised if you were a diabetic or if you have a record, medical diagnosis or diabetes. So, the test strips are heavily subsidised and that’s where the companies make their big money so they’re very, very interested in giving you the monitor so you would the test strips. Because if you bought them independently, I think they are 50, 60, maybe $70 a packet. And I get them for $5 something, $6 but they’re getting 50, $60 a box of tests. So, they want you to use their things.

P14, Nancy

Furthermore, 8 (36%) participants received help or technical support for the technologies from government or technology providers. One participant stated the following:

Well, the hearing aids are the ones that the government provides for pensioners, so there’s no financial support except I pay for batteries and having them checked up. Once a year I go for a checkup with them. The pendant is...What’s it called? Well they do come, supposed to come once a year and check them, and once a month I ring the alarm just to see that it’s working.

P3, Cyndi

In addition, 2 (9%) participants mentioned that their living arrangements provided support for their needs that enabled independent living. That is, they saw the value of technologies for supporting independent living. One lived in a retirement village and the other in public housing (affordable government-owned homes that support older adults with accommodation issues). Both participants moved into homes that offered support and immediate assistance through technologies for health self-management. One participant who had a chronic illness and disability and lived in public housing said the following:

I love my little home and this chair and my computer and easy cooking and all of those things…And they did an upgrade of the units two and a half years ago, and they absolutely focused on my disability needs…I’ve got an oxygen concentrator here, I’ve got P2.5 masks and everything has been done to enable my functioning…all those fine details which the number of faxes and emails for my doctors to get everything that we reckoned, this was a one-off change to get this unit right for my aging, more assistive technologies [scooter, walking stick, pickup stick, orthopaedic lift up chair, and push along walker].

P22, Zoe

Another participant moved to a retirement village because of her husband’s health issues and the need to access support:

The pendant alarm is part of being in the village, and that was one of the reasons we moved here, to have that sort of backup after my husband’s brain tumor. We didn’t know what the future meant for him, so that’s been handy because he was recently ill, and pressed the pendant, and the ambulance was here straight away.

P19, Tina

#### Theme 5: Technology Provided Convenient Access to Health Care and Safety

In total, 13 (59%) out of the 22 respondents commented that technology such as telehealth was convenient and enabled prompt medical attention. The technology provided efficiency benefits because it reduced travel and wait times at hospitals for the participants:

It wasn't really very much available before, but I will continue to use it now, because there’s no point driving out to the doctor if you don’t need to see him. It takes less time for the doctor to see you on telehealth and less of your time as well.

P1, Amy

Furthermore, 3 (14%) participants who needed support mentioned that older adults who could not move easily or needed to stay home could benefit from telehealth because of its convenience and easy access to medical staff. As one participant noted, telehealth improved access to health services:

Telehealth, I think telehealth for some people is really good, and at times for old people because sometimes you just can’t get somewhere. So being able to have sort of a consult with a doctor but not a situation where it’s five minutes or 10 minutes and you’re in and out, but a proper consult with your own doctor can be great at those times when people are housebound where they can’t move easily and can’t get out.

P8, Helen

Overall, 8 (36%) participants, including 3 (38%) of the 8 who were living alone and needed support, said that they used technologies such as pendant alarms because they offered safety and access to services and support. This enabled the participants to feel comfortable living independently:

The pendant because I live alone and I do a few risky things like climbing ladders, so in case I have a fall I’ve got someone I could contact. Otherwise, I’d be on my own without help.

P3, Cyndi

#### Theme 6: Easy Setup and Low Cost of the Technology

In addition, of the 22 participants, 7 (32%) commented that the technologies that they were using to monitor their health were easy to set up and use. A participant who was using apps related to fitness said the following:

No, there’s nothing I don’t like about it [related to the app]. I found it easy to use. No, there’s nothing I don’t like about it. It serves its purpose for me so I like it.

P7, Gwen

Of the 22 participants, for 4 (18%) of them, cost was not an issue to obtain and use technology. A participant commented that the price to buy technologies for health self-management such as blood pressure monitor, pendant alarm, pulse oximeter, and telehealth was affordable:

I haven’t sought financial support, because they’ve all been within a reasonable price bracket.

P17, Rita

### Barriers and Challenges: Factors that Discourage the Use of Technologies for Health Self-management

#### Overview

Our analysis identified 4 barriers and challenges that interviewees had encountered when using technologies for monitoring their health. Barriers include information overload and a sense of futility about future health decline; telehealth being an inadequate substitute for in-person consultation; concerns about trust related to privacy and accuracy; and technologies being stigmatizing, uncomfortable to use, expensive, and unfamiliar.

#### Theme 7: Information Overload and Sense of Futility About Future Health Decline

Of the 22 participants, 10 (45%) were motivated to manage their health because of the risk of developing certain conditions. There were 2 (9%) participants, however, who appeared to feel overwhelmed by gaining too much knowledge about their existing conditions from using the technologies. This caused them distress and anxiety. For example, a participant who was using devices such as a blood pressure monitor, blood glucose monitor, and a fitness tracker to control diabetes said the following:

You’re a diabetic whose weight has got high glucose level but weight now follows your need to exercise more, but you’ve got this pain. So what can we do first? Are there any signs that we need to change to diet? And what are you prepared to do? But it just gets more difficult the more things you have to think about.

So I think people go for the easy stuff and I’m just trying to think, “All right. Try not to think about the cardiovascular stuff. I didn’t need to know that. I really did not need to know that.” The arthritis and the diabetes is enough, thank you and COVID made me very anxious, so I was very anxious.

P14, Nancy

Another participant, who had hyperacusis and was unable to tolerate loud sounds, commented that she received news about possible complications owing to the use of hearing aids and she had to decide whether she would be deaf or experience the complications of using hearing aids. This information caused her more distress because she had already been dealing with other health issues:

Well what are normal sounds for most people, cars going along the street and so on, or a dog barking. She [neurologist] said, ‘I’ve had to set your hearing lower than I’d like because of all of that.’ But the sound was too much. So we’re in the trial stage and you know I might have to make the choice, okay I’m just going to get deaf and I might make the choice that I’m going to struggle with this. But I’m going to get brain symptoms if I use them [related to hearing aids]. My eyes are tearing up as I say all of that. It’s very hard.

P22, Zoe

Of the 22 participants, 5 (23%) expressed a sense of futility about future health decline stating that no matter what they did, they could not avoid inherited or other diseases. One participant who used a blood pressure monitor and a fitness tracker said that she was concerned that she would still have cardiovascular disease, as her family members had before her:

Well, unfortunately my genetic background is full of heart trouble. So I presume I will end up with heart problems. Other than that, there has been cancer in the family, but not immediate family. So hopefully there would no problem there. And someone said as we get older, the worry is that you fall over and that could be a problem as well.

P21, Whitney

The participants commented that no matter what they did to be healthy, as they got older, they would become more fragile and more prone to disease, and something unexpected could happen:

And as you get older you’re going to expect to get more frail, and you’ll probably get cancer or something at some time, but that’s life. Who knows what’s going to front up, but when you get to 75, you’ve got max 25 years left, so you’re going to pop off sometime.

P10, John

#### Theme 8: Telehealth Cannot Fully Replace In-Person Consultations

Some participants commented on the limitations of telehealth. In total, 4 (18%) participants communicated with physicians through phone calls, whereas others (5/22, 23%) communicated by Zoom. However, patients had to visit the hospital when physical examination or immediate attention was necessary. A participant who had osteoarthritis and mild sleep apnea and had consulted with the physician over the phone commented the following:

So, in the last nine months I would say, I’ve used a combination of...sometimes I’ve had to go to the doctor’s in person for an injection right? You can’t do that with Telehealth. But other times I’ve been able to have a consultation over the phone.

P18, Sarah

In addition, the participants mentioned that there was the lack of interaction and connection with the medical staff. The participants had used telehealth through their phones, and they could not interact with the physician. Therefore, they preferred face-to-face interactions:

But with telehealth, I understand that it’s necessary to do because you can’t have personal contact, but for me, if you can’t have a personal dialog with your medical practitioner, it’s not quite the same thing, I don’t think…If you don’t have that connection with the eyes that you understand exactly what they mean, because sometimes a doctor can say to you, ‘That’s really what I need you to do, do you understand?’ And sometimes you do, but sometimes they may mean…something a little bit more dramatic that the very serious…So look, in the telehealth that I’ve been doing, it’s been by phone…So there’s been no visual…So that’s been unsatisfactory. But I like the doctor and so I’ve still tried to do the best you can.

P9, Isaac

#### Theme 9: Concerns About Trust Related to Privacy and Accuracy

Security and privacy implications were mentioned by 4 (18%) of the 22 participants. The participants were concerned about privacy related to providing data for data collection, sharing information, and avoiding identity theft from technologies such as fitness trackers, apps, videoconferencing software, and messaging apps:

I guess the privacy concerns are just the suggestions are that the old people were worried about rumors and suggestions of people intruding on identity theft of getting knowledge about you in certain ways. And also of course, I imagine you understand the words, scams, the sort of…that sort of thing, yes.

P16, Paul

I don’t join my Fitbit up to the…anything else. I refuse to give away my analog information. I don’t know where it’s going, so I don’t have it joined up to the app. I’ve turned the app off so that it just gives me numbers and I charge.

P14, Nancy

In addition, 3 (14%) participants reported the inaccuracy of the measurement of the blood pressure monitor:

I am, just because of my physics background, suspicious of their accuracy, which as I said, I had three [related to blood pressure monitor], and I’ve taken measurements from using all three in quick succession, or simultaneously, and then cross checked the results, so I always have a degree of skepticism about their precision.

P10, John

#### Theme 10: Health Care Technologies Can Be Stigmatizing, Uncomfortable to Use, Expensive, and Not Familiar

Of the 22 participants, 2 (9%) participants expressed concerns about the visibility of the device and the stigma associated with using health care devices such as pendant alarms. The participants believed that they were wearing a technology that targeted older adults with frailty. By using these technologies, other people could perceive them as frail or with poor health condition. For example, one participant commented as follows:

I remember my late mum she used to have to wear something around her neck which she hated 24/7 and felt like a cow. She really hated that and I can totally relate now. So we were talking about devices that might help us, like something that we could wear on our wrist perhaps the Apple Watch for example or some other device that was on our wrist that was not intrusive and didn’t look awful and identify us as being in a certain age group and having certain health problems and all of those types of things. So something like that definitely we would both look into it.

P8, Helen

Furthermore, 4 (18%) participants reported discomfort when using health-related technologies such as CPAP machine, blood pressure monitor, hearing aids, and implantable cardiac defibrillator. A participant who had sleep apnea mentioned that she felt uncomfortable while using it:

…And I mean, with things like C-PAP machine, no one in their right mind would get one if they didn’t need one, because it’s hideously, intrusive and uncomfortable. It’s awful to have to use. So if I had a choice I wouldn’t use it ever.

P15, Olivia

In addition, 1 (5%) participant expressed concerns about the cost of technologies:

So, for example, with a C-PAP machine, cause I’ve used one for about 10 years, I think and I’m on my second machine. They have a lifespan of about five, six years maybe. They’re expensive. They’re very expensive in Australia. If I lived in the US I’d get one for a third of the price, even from a company that’s designed it in Australia. So really pisses me off that for some things where you really have little choice, you’re not supported through Medicare [Australian public health insurance system], but you need these things and yet you’re talking thousands of dollars. So if they need a service, that can be a thousand dollars. So I suppose like with any technology I suppose the thing that frustrates me with them is sometimes the need for maintenance is high and there’s a cost associated with that.

P15, Olivia

Furthermore, 2 (9%) participants expressed an issue regarding the battery of the devices that drained quickly. A participant mentioned the following:

The costs of charging. You seem to always...have you got it charged, or haven’t you got it charged, and that’s probably the most annoying thing. And if you go anywhere, you seem to take more chargers than anything with you.

P19, Tina

In addition, 3 (14%) participants also shared concerns about their lack of familiarity with the technology:

The only thing I would say is, although most older people are quite tech savvy now, there’s still a lot of people who don’t have a computer. So there’s still that gap…

So, for example, I’m president of a group and there’s about 15 people in it. And several of them I have to communicate with them either by going in person or by writing to them snail mail. Or by ringing them up because that’s what I can do with them.

And with others, I can quickly either send a text because lots of people got phones, but not everybody knows how to text.

P18, Sarah

## Discussion

### Principal Findings

This study aimed to understand what types of technologies older adults use to support their health, what motivates older adults to use technologies to support their health, and what factors enable the effective use of technologies for health self-management. Finally, this study investigated the barriers that negatively affect the adoption and use of technologies for health self-management.

A range of communication technologies such as videoconferencing software or phone calls were most commonly used to enable telehealth consultations. Wearable devices, medical devices, and web-based sources for health information were also discussed. Motivators that encouraged participants to use technology included a desire to monitor their health, stay active and connected, and monitor health signs, especially when they were already aware of a personal risk related to future health decline. Enablers that facilitated the positive uptake of technologies for health self-management included social and organizational influence, technology-enabled convenient access to health care and safety, and easy setup and low cost of the technology.

However, our findings showed that there are challenges that affect the use of technology by older adults. These include feeling overwhelmed by too much information; feelings of helplessness about future health decline; telehealth being a poor substitute for in-person consultation; concerns about trust in system privacy and accuracy; and technologies being stigmatizing, uncomfortable to use, expensive, and unfamiliar.

In the following sections, we discuss the importance of our findings in relation to previous work and present further interpretations.

### Motivators to Use Technology: Planning for Resilient Aging

We found 3 main motivators for using technologies for health management among our participants. Drawing on these findings, we argue that the knowledge of future adversities provided a sense of power and control for some of our participants. They expressed attitudes toward aging that demonstrated personal resilience and used technologies for health self-management to prevent or prepare for future health decline.

These findings can be interpreted using the lens of resilient aging. Resiliency is defined as the ability to recover from difficult situations, that is, “an extraordinary atypical personal ability to revert or ‘bounce back’ to a point of equilibrium despite significant adversity” [32]. Adversity can be viewed in terms of living conditions that lead to personal losses, inequalities, disabilities, and general challenges of aging [33].

Researchers have identified the ways in which systems can be designed for future resilience. Our findings suggest that participants have different approaches to preparing for future resilient aging. This points to a type of resilience that is related to the definition provided by Woods [34]: “to be resilient, a system looks ahead to read the signs that its adaptive capacity as it currently is configured and performs is becoming inadequate to meet the demands it will or could encounter in the future.” According to Hollnagel [35], the fundamental functions for resilience include anticipating and monitoring changes and threats, being proactive, ensuring the ability to respond to interruptions, and learning from past experiences.

We observed this pattern of resilience in some of our participants. Knowledge of future adversities provided them with a sense of power and control, and knowledge about what to expect in the future meant that they knew what to do about their health and could make decisions with this knowledge in mind. For example, some participants, who monitored their health because of their family history, took early action and planned ahead to avoid or delay the appearance of hereditary diseases. Dismissing information about their health and not doing something about it could make them more susceptible to health decline and the loss of independence. A previous study reported that risk factors for the loss of independence in later life include poor mental and physical health, social isolation, the loss of mobility, inappropriate environment and living conditions, and the lack of resources [36]. Thus, older adults are likely to benefit from using technologies that allow them to maintain their independence; give them control and authority over the characteristics and functions of the technologies; and do not make them appear weak, dependent, or in need of special care [37].

In contrast, other participants showed less resilience. For these participants, this knowledge could cause anxiety and stress, as this information overwhelmed them and they did not know what to do. That is, information about the deterioration of their health created a feeling of helplessness in some participants. Previous research found that information overload creates stress, fatigue, burnout, and even interruption in the use of information sources [38,39]. In addition, information overload negatively affects psychological well-being and influences the intention to discontinue the pursuit of health information [39] and results in information avoidance [38].

We argue that participants who chose technology to track health and activity information were those who expressed a resilient attitude by preparing in advance to prevent or delay the onset of hereditary diseases. In contrast, those participants who said they felt overwhelmed with the information provided about their existing medical conditions were using technology to manage their health. This leads us to argue that the link between resilience and technology use for health self-management warrants further investigation.

### Enablers to Use Technology: Social and Organizational Influence Is the Most Mentioned

This study identified 3 enablers in using technologies for health management among our participants.

First, our findings showed that social and organizational influence positively affected technology use. All the participants mentioned the influence of family and friends, health care staff, and organizations. In this study, social and organizational influence provided information about health-related technologies and helped in motivating the use of these technologies. In addition, some technologies were subsidized, and older adults received help or technical support from nonprofit organizations related to older adults; public and private organizations that research and assist people with diabetes, tinnitus, hearing loss, and dementia; or technology providers. Social and organizational influence became enablers and positively affected the use of technologies when they helped overcome technology barriers. In contrast to the Technology Acceptance Model [40] and the Unifying Theory of the Acceptance and Use of Technology [41], we observed clear evidence that social and organizational factors not only influenced the intention to use technologies but also shaped how they were used in practice. The influence of social context on actual use is not found in either model, so capturing this would be a vital element in a framework that captures the use of supportive technologies by older adults.

Previous research showed that older adults who received support in addressing technological challenges were more willing to use various products and devices in their daily lives [42]. Family members, friends, and medical staff often comment and recommend recent technology to older adults, and this influences older adults’ technology use decisions [42]. According to Tseng et al [43], the opinions of other older adults can influence older adults’ acceptance of the health monitoring system. On the basis of this finding, influential people such as health care professionals, family, and caregivers could be included in the design of the technologies for health self-management because they are familiar with the technology owing to the assistance they provide to older adults.

In addition, we found that organizational influence such as supported living arrangements influenced the participants’ decisions about using technology. These accommodations offered support and prompt assistance to the participants through technologies for health self-management. Consistent with these findings, previous research has found that older adults at risk of losing their independence will try to adjust to their environment, such as finding suitable housing that will allow them to carry out the daily activities necessary to maintain their independence [36].

Focusing on the second enabler, we observed that our participants found technology such as telehealth to be convenient, enabling them prompt access to medical attention. In addition, participants who needed assistance mentioned that older adults who could not move around quickly or needed to stay at home might benefit from telehealth owing to its convenience and easy access to medical staff. In addition, the technology demonstrated efficiency benefits because it reduced travel and wait times at hospitals for the participants. Furthermore, participants who lived alone and required assistance mentioned using technologies such as pendant alarms because they provided safety and access to services and support. This finding supports the evidence from a previous study in which participants mentioned the benefits of telehealth, including convenience and cost [26]. In addition, participants reported that telemedicine health services were convenient because they eliminated travel and waiting times, saved money, and allowed them to complete the consultation from the comfort of their homes at any time [44]. In addition, results related to the safety provided by the technology are consistent with the studies in which participants reported a sense of safety and security because of using technologies, as well as a desire to use technologies to prevent or detect accidents and medical emergencies [45,46].

Finally, participants stated that the technologies that they were using to monitor their health were simple to set up and use and affordable. In a systematic review of web-based home consultation platforms, Almathami et al [26] found that the ease of use related to the ease of navigation and use of services and savings, based on the cost of mileage traveled per patient.

In this study, the enabler that was the most mentioned by the participants was the social and organizational influence. All the participants mentioned the influence of family and friends, health care staff, or organizations in their use of technologies. Therefore, we can conclude that social and organizational influence can play an important role in determining whether and how older adults will use health care technologies.

### Perceived Need Trumps Barriers and Challenges

We found that our participants faced similar barriers to technology adoption as reported in previous studies related to using home health care technology such as wearable devices, smart home technologies, telemedicine, and other technologies that help older adults remain at home [24,47-56].

One of the barriers is that some participants felt overwhelmed by the information provided by the technologies related to their existing health conditions. This information overload caused anguish and anxiety. This finding broadly supports studies related to mobile health services and glucose monitoring, and they state that information and system feature overload increased older adults’ fatigue and stress, thereby increasing their resistance to the adoption of these technologies [57,58].

One finding concerns the limitations of telehealth, such as the lack of physical examination or immediate attention and lack of interaction and connection with the medical staff. This finding accords with that of previous studies, in which participants expressed an interest in connecting with their health care provider for the want of human interaction [59] and a perceived lack of care integrity when care was delivered through video visits [60].

Our findings also highlighted older adults’ concerns about trust related to privacy and accuracy. These results corroborate the findings of the previous work of LaMonica et al [61], who observed that data privacy and security risks were primary barriers to health technology use. If the digital technology is provided by reputable sources such as health organizations, universities and academics, and individuals with higher degrees or qualifications, these barriers could be mitigated. In addition, some studies related to monitoring systems or electronic health records have revealed that older adults have concerns about the privacy and confidentiality of their health information [49,50,62]. This is challenging to overcome, as the purpose of these systems is to collect and share their data, and these concerns could influence their willingness to adopt and use them.

In addition, some studies showed that older adults with greater concern for privacy will choose human support over health information technology when they are given the option, and older adults with disabilities are willing to give up their privacy for independence, but they need to make informed decisions [63].

Another barrier is that health care technologies can be stigmatizing. This finding was also reported by Mitzner et al [64] and Demiris et al [24] who showed that the fear of stigma can prevent older adults from embracing and using technology. According to Blythe et al [65], a design with the potential to stigmatize its users reinforces a particular view of older adults’ place in society. For example, studies have revealed that wearable sensor devices or personal emergency alarms have a negative image among older individuals because they are conspicuous, identifiable as a care item, and even humiliating [66,67]. The fear of stigmatization or of being labeled as disabled or sick influences the adoption and use of health care devices. Stigma may become less of a concern for older adults when the need to use health care technologies becomes urgent [62]. Therefore, older adults are more likely to adopt technologies that they do not view as stigmatizing, such as smart watches, which are widely used and not immediately identifiable as a health care device.

Our result related to the cost of the technologies is consistent with that of previous studies that have mentioned cost as a barrier to overcome and affect the use of technologies to assist health care such as health information and assistive technologies [46,50,62,66,68]. It is important that technologies are affordable; otherwise, older adults would drive away from using technology [37].

The lack of familiarity is another barrier mentioned. These findings support evidence from a previous study on health information technologies such as telecare, electronic health record, decision support systems, and assistive information technologies, which found issues with familiarity as a barrier that older adults face when using technology [63]. In addition, some studies showed that some older adults with minimal technology experience prefer to rely on health care providers and carers for health tracking [69,70].

Our last finding related to perceived need supports evidence from previous studies, in which perceived need for the technology is a variable that could influence the adoption of new technologies [23,25,71-73], and it has been shown to be a significant factor in the acceptance of assistive technologies [74]. However, our findings do raise the question of why older people continue to use technology despite the presence of barriers. One reason is that the perceived need trumps the barriers to using technologies. Our participants were using health care technologies because there is a perceived need that motivates them to overcome perceived barriers. For example, even if they feel overloaded with information, they will continue to use technology because it helps manage their health. Although this suggests that older adults will tolerate these barriers if the need outweighs them, technology designers should focus on alleviating barriers to promote the uptake of technologies for health self-management by older adults.

### Limitations and Future Work

One limitation of our study is the wide age range of the participants, spanning 20 years. This included, presumably, recent retirees (ie, those in their 60s) and those approaching advanced age. There is a significant difference between these 2 groups regarding needs and capabilities.

Second, most participants (20/22, 91%) had tertiary education, and this is not a representative sample. Previous studies have shown that a person’s level of education was significantly associated with technology acceptance [75-77]

A third limitation is that this study provides an overview of factors but does not differentiate these factors between types of health self-management technologies. Therefore, the results may not be specific for each technology mentioned in this study.

Finally, the data for this study were collected during the COVID-19 pandemic. Owing to the restrictions imposed to prevent the spread of the virus, older adults had to access telehealth through videoconferences or consultation calls to access medical care [78]. Telehealth was the most common technology used by the interviewees, and some barriers related to the use of telehealth prevailed. Future studies could be carried out in nonpandemic times and could perhaps highlight other findings related to the barriers to using and accessing technologies other than telehealth.

### Conclusions

This study investigated the use of digital technologies for self-management of health by older adults. On the basis of the range of technologies that support older adults’ health, we argue that some participants showed a resilient attitude, taking early measures to monitor their health and stay active, for which they were more willing to use technology. In addition, we argue that older adults’ perceived need outweighs technological barriers, so they will continue using the technology if it gives them value. We also found social and organizational influence to be one of the most mentioned enablers of the use of technologies.

The analysis of the interviews provides useful information for the design and implementation of future technologies for the self-management of health. Future studies could explore resilience, as this study shows evidence related to resilience that could influence the use of technologies for health self-management. In addition, we argue that it may be necessary to help people gain resilience in the face of future health decline before introducing health care technologies.

Finally, technology providers could investigate the social and organizational influence and incorporate their findings in technology design as this factor affects the adoption of technologies. They could also explore older adults’ motivations to use technologies and reduce the barriers that they face.

## Appendix

Multimedia Appendix 1 Interview participants.

## Abbreviations

CPAP: continuous positive airway pressure
