# Supplementary material for: Understanding Older Adults’ Experiences With Technologies for Health Self-management: Interview Study
Source: JMIR Aging. 2023 Mar 21;6:e43197. doi: 10.2196/43197 (PMC10131633; doi:10.2196/43197)
Supplement: Multimedia Appendix 1 [file aging_v6i1e43197_app1.docx]

Multimedia Appendix 1. Interview participants

| **No.** | **Nickname** | **Internet connection at home** | **Age** | **Gender** | **Highest level of education** | **Marital status** | **Working or retired** | **Household composition** | **Need for help or supervision** | **Reasons for help or supervision** |
| --- | --- | --- | --- | --- | --- | --- | --- | --- | --- | --- |
| P1 | Amy | Yes | 71 | Female | Tertiary institution/University or other higher educational institution | Married | Retired | Live with her partner | No | No need for help or supervision |
| P2 | Bob | Yes | 80 | Male | Tertiary institution/University or other higher educational institution | Married | Semi-retired | Live with his partner | No | No need for help or supervision |
| P3 | Cyndi | Yes | 82 | Female | Tertiary institution/University or other higher educational institution | Single | Retired | Live alone | No | No need for help or supervision |
| P4 | David | Yes | 87 | Male | Secondary school/Government | Widowed | Retired | Live alone | Yes, sometimes | Short-term health condition (lasting less than six months) |
| P5 | Elvis | Yes | 73 | Male | Tertiary institution/University or other higher educational institution | Married | Retired | Live with his partner | No | No need for help or supervision |
| P6 | Frank | Yes | 66 | Male | Tertiary institution/University or other higher educational institution | Married | Retired | Live with his partner | No | No need for help or supervision |
| P7 | Gwen | Yes | 65 | Female | Tertiary institution/University or other higher educational institution | Married | Retired | Live with her partner | No | No need for help or supervision |
| P8 | Helen | Yes | 68 | Female | Tertiary institution/University or other higher educational institution | Single | Retired | Live alone | Yes, sometimes | Long-term health condition (lasting six months or more) |
| P9 | Isaac | Yes | 66 | Male | Secondary school | Single | Retired | Live with his partner | No | No need for help or supervision |
| P10 | John | Yes | 75 | Male | Tertiary institution/University or other higher educational institution | Widowed | Semi-retired | Live alone | No | No need for help or supervision |
| P11 | Katy | Yes | 86 | Female | Tertiary institution/University or other higher educational institution | Widowed | Retired | Live alone | No | No need for help or supervision |
| P12 | Linda | Yes | 69 | Female | Tertiary institution/University or other higher educational institution | Married | Retired | Live with her partner | No | No need for help or supervision |
| P13 | Michael | Yes | 66 | Male | Tertiary institution/University or other higher educational institution | Married | Retired | Live with his partner | No | No need for help or supervision |
| P14 | Nancy | Yes | 74 | Female | Tertiary institution/University or other higher educational institution | Married | Retired (retired because of COVID) | Live with her partner | No | No need for help or supervision |
| P15 | Olivia | Yes | 70 | Female | Tertiary institution/University or other higher educational institution | Married | Working part-time. | Live with her partner | No | No need for help or supervision |
| P16 | Paul | Yes | 73 | Male | Tertiary institution/University or other higher educational institution | Married | Retired | Live with his partner and son | No | No need for help or supervision |
| P17 | Rita | Yes | 68 | Female | Tertiary institution/University or other higher educational institution | Married | Retired | Live with her partner | Yes, sometimes | Long-term health condition (lasting six months or more) / long term disability. |
| P18 | Sarah | Yes | 77 | Female | Tertiary institution/University or other higher educational institution | Divorced | Retired | Live alone | No | No need for help or supervision |
| P19 | Tina | Yes | 72 | Female | Tertiary institution/University or other higher educational institution | Married | Retired | Live with her partner | No | No need for help or supervision |
| P20 | Vera | Yes | 71 | Female | Tertiary institution/University or other higher educational institution | Married | Retired | Live with her partner | Yes, sometimes | Other cause/community services come in to clean. |
| P21 | Whitney | Yes | 77 | Female | Tertiary institution/University or other higher educational institution | Widowed | Retired | Live alone | No | No need for help or supervision |
| P22 | Zoe | Yes | 76 | Female | Tertiary institution/University or other higher educational institution | Prefer not to answer | Retired | Live alone | Yes, sometimes | Chronic illness and disability |
